# An appetite for growth: The role of the hypothalamic – pituitary – growth hormone axis in energy balance

**DOI:** 10.1111/jne.13133

**Published:** 2022-04-26

**Authors:** Rebecca Dumbell

**Affiliations:** ^1^ School of Science and Technology, Department of Biosciences Nottingham Trent University Nottingham UK

**Keywords:** adiposity, bodyweight, exercise, IGF1, photoperiod

## Abstract

Links between the regulation of growth and energy balance are clear; to fuel growth, there must be consumption of energy. Therefore, it is perhaps intuitive that interactions between the hypothalamic – pituitary – growth hormone axis (growth axis) and pathways that drive metabolic processes exist. Overproduction of growth hormone has been associated with diabetes and metabolic disease for decades and the opposing effects of growth hormone and insulin have been studied since early experiments almost a century ago. The relationship between neuroendocrine axes can be complex and the growth axis is no exception, interacting with energy balance in several organ systems, both in the periphery and centrally in hypothalamic nuclei. Much is known about peripheral interactions between growth axis hormones and processes such as glucose homeostasis and adipogenesis. More is still being learned about the molecular actions of growth axis hormones in adipose and other metabolically active tissues, and recent findings are discussed in this perspective. However, less is known about interactions with central energy balance pathways in the hypothalamus. This perspective aims to summarise what is known about these interactions, taking lessons from human studies and animal genetic and seasonal models, and discusses what this may mean in an evolving landscape of personalised medicine.

## 1 INTRODUCTION

Growth hormone (also called somatotropin) is a pleiotropic hormone that was first isolated from bovine pituitary glands as a crude extract in the 1920s and demonstrated to accelerate growth in different model species. In humans, disruption of the hypothalamic – pituitary – growth hormone axis (growth axis) occurs as a consequence of over production of growth hormone and downstream hormones often caused by pituitary tumours in pituitary gigantism or acromegaly (occurring before or after epiphysial fusion of the long bones respectively) or by congenital or acquired growth hormone deficiency.

**FIGURE 1:**
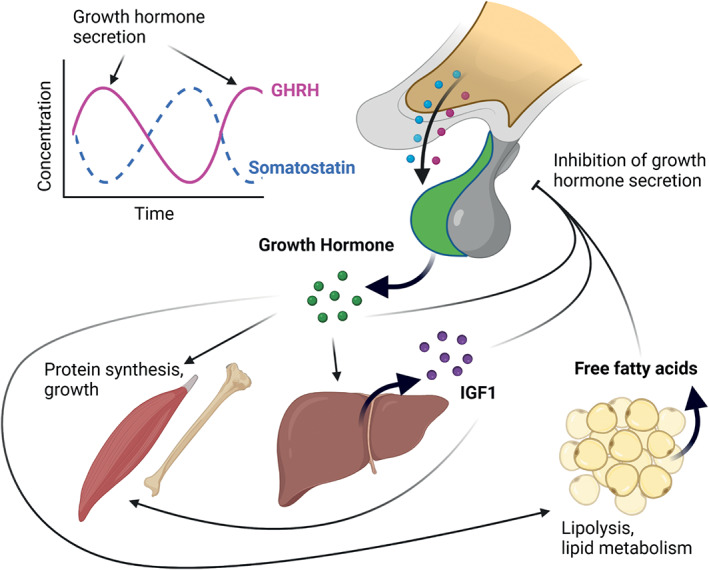
The regulation of pulsatile growth hormone secretion starts at the hypothalamus with growth hormone‐releasing hormone (GHRH) and somatostatin secretion from the arcuate nucleus (ARC) and periventricular nucleus (PeVN) respectively. Growth hormone is secreted from the somatotrophs of the anterior pituitary and stimulates anabolism and growth in multiple tissues both directly and through insulin‐like growth factor‐1 (IGF1), released from the liver. In adipose tissue growth hormone is catabolic, stimulating lipolysis and liberation of free fatty acids to drive lipid metabolism. Growth hormone, IGF1 and free fatty acids all inhibit release of growth hormone, either directly at the pituitary or by stimulating release of somatostatin from the PeVN

As a neuroendocrine pathway, important components of the growth axis reside in the hypothalamus. Growth hormone is released from the anterior pituitary, stimulated by growth hormone releasing hormone (GHRH) and inhibited by somatostatin (also called *somatotropin‐release inhibiting factor*, SRIF). GHRH and somatostatin are secreted from arcuate nucleus (ARC) and periventricular nucleus (PeVN) neurons of the hypothalamus respectively and reach the anterior pituitary through the hypophyseal blood portal system and median eminence. Pulsatile secretion of growth hormone from the somatotrophs of the anterior pituitary peaks in both amplitude and frequency during the night, and the amplitude of growth hormone pulses increase with age until puberty, when growth is at its most rapid.^1^ Growth hormone acts through the growth hormone receptor, a cytokine class I receptor, which is broadly expressed throughout the body, and whose actions are mediated by JAK/STAT signalling (recently reviewed in^2^). The growth axis is an unusual endocrine axis, in that some of the growth effects are then mediated by a further hormone – insulin‐like growth factor‐1 (IGF1), produced in the liver and with a significantly longer half‐life than growth hormone. However IGF1 is not always required for the somatic growth action of growth hormone. Both growth hormone and IGF1 stimulate somatic growth and exert negative feedback on the growth axis by increasing secretion of somatostatin in the periventricular nucleus (PeVN) of the hypothalamus (Figure 1). Interestingly, a proposed growth axis mediated “gravitostat” has been hypothesised – a bodyweight sensing mechanism during adulthood and in puberty. Rats implanted with weighted capsules to the abdomen slowed weight gain and underwent catch‐up growth when removed.^3^ This was accompanied by altered hypothalamic *Ghrh* expression and serum IGF1 levels, suggesting altered growth axis negative feedback.

The main effects of growth hormone and IGF1 are to stimulate somatic growth, with the notable exception of adipose tissue, where its action is catabolic. Growth hormone acts to cause lipolysis and increase availability of fatty acids for lipid metabolism, and therefore has opposing effects to insulin. This means it has an important role to play in peripheral metabolism.

More recent work has demonstrated different central interactions for the hormones of the growth axis, to regulate food intake, energy expenditure and potentially influence macronutrient preference in the diet. This perspective will summarise what is known about the action of the growth axis in the periphery and centrally to influence energy balance, highlighting exciting new developments and insights from studies in seasonal mammals that display annual cycles in growth and metabolic phenotype. This will be discussed in the context of the emerging concept of personalised medicine, and where future investigations harnessing new technologies may lead.

## 2 THE GROWTH AXIS AND PERIPHERAL METABOLISM

The growth axis has a role to play in adipose tissue function. Growth hormone is lipolytic, and lessons from mouse genetic models tell us that increasing growth hormone in circulation often leads to decreased fat mass. Mouse models of the growth axis have recently been thoroughly systematically reviewed.^4^ Early mouse lines with spontaneous mutations in growth axis components have existed as early as the 1920s, and in general mice overexpressing growth hormone are larger, with increased circulating growth hormone and IGF1 levels and have decreased insulin sensitivity and decreased adiposity. The opposite is true for genetically altered mice or those with a spontaneous loss of function mutation leading to decreased circulating growth hormone and IGF1. These mice have a short body length, associated with decreased growth, improved insulin sensitivity and relatively increased adiposity.

**FIGURE 2:**
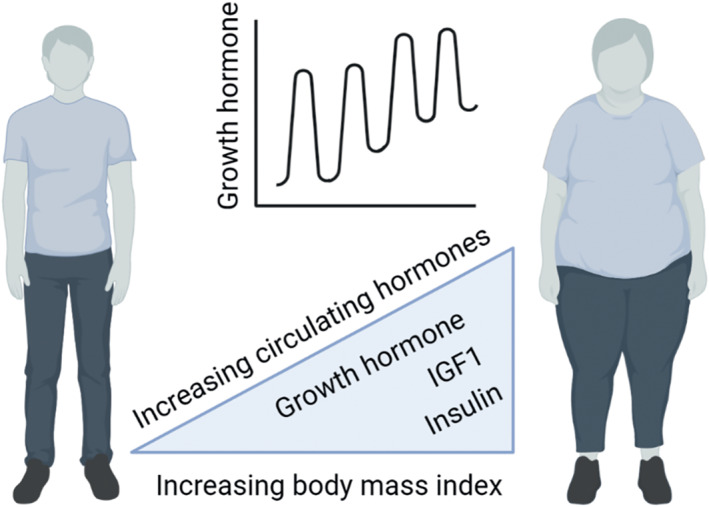
Human plasma levels of growth hormone, insulin‐like growth factor‐1 (IGF1), and insulin are all positively correlated with body mass index, as are higher trough levels of pulsatile growth hormone secretion

The nature of pulsatile growth hormone secretion is also a determinant of body mass and fat distribution in humans. Lower inter‐pulse growth hormone levels are associated with lower body mass index and lower waist‐hip‐ratio^5^ – indirect indicators of adiposity and body fat distribution, respectively and levels of IGF1 and growth hormone negatively correlate with adiposity in obese humans and rodents^6^, ^7^ (Figure 2).

The action of growth hormone is generally considered diabetogenic and acutely stimulates lipolysis and increased free fatty acids in circulation following growth hormone administration. This effect was demonstrated as early as the 1930s with cats and dogs treated with pituitary extracts. People with acromegaly or pituitary gigantism have an overproduction of growth hormone and IGF1 and are more likely to suffer from diabetes (reviewed in^8^). Work in the 1980s demonstrated that increased circulating free fatty acids that result from lipolysis provide some measure of negative feedback to inhibit growth hormone secretion, probably by increasing somatostatin release.^9^ As discussed further below, more recent work in in vitro and in transgenic mice demonstrates that growth hormone acts at adipocytes both to promote adipogenesis,^10^ but also suppressing expression of key regulators of adipogenesis fat‐specific protein 27 (FSP27) and peroxisome proliferator‐activated receptor gamma (PPARY) (reviewed in^11^).

In mouse models, adipose tissue selective knockout of the growth hormone receptor, driven by either an *Fabp4 Cre* ^12^ or *Adipoq Cre* targeting mature adipocytes^13^ both result in increased overall fat mass in experiments from the same group. The effect of IGF1 receptor knockout in adipose tissue has been investigated using the same two CRE drivers and resulted in opposite effects on overall fat mass; with the *Fabp4 Cre* driven knockdown having greater fat mass^14^ and the *Adipoq Cre* driven deletion having less fat mass than wild‐type littermates.^15^ This discrepancy is probably explained by *Fabp4* expression in additional cell types such as skeletal muscle, endothelial cells, and macrophages, leading to loss of IGF1 signalling in other metabolically important tissues. The diabetogenic action of growth hormone is not only through disrupted adipose physiology. Since growth hormone has opposing effects to insulin in adipose tissue, over‐secretion of growth hormone leads to insulin resistance. However, growth hormone also promotes insulin resistance in other metabolically active tissues through multiple mechanisms such as disrupted insulin signalling, and triglyceride build up in the liver and skeletal muscle (reviewed in^11^). Together these studies demonstrate an important role for growth hormone and IGF1 in peripheral metabolism and glucose homeostasis, demonstrating an important role in the development of metabolic disease.

## 3 THE GROWTH AXIS IN THE HYPOTHALAMUS

The pulsatile release of growth hormone from the pituitary is generally understood to be regulated by pulsatile secretion of GHRH from ARC neurons when somatostatin secretion from the PeVN is low.^1^ These neuropeptides are released from neurons in the ARC and PeVN that project to the median eminence to reach the anterior pituitary somatotrophs through the hypophyseal blood portal system.^16^, ^17^, ^18^ However, somatostatin neurons are also present in the ARC and their function is more elusive. Somatostatin neurons in the ARC do not appear to be responsive to growth hormone (reviewed in 19), and they mainly project to GHRH neurons within the ARC.^17^, ^20^ It is therefore likely that the role of ARC somatostatin is to regulate GHRH release directly. This is supported by a recent immunohistochemical study in human brain tissue where ARC somatostatin neurons appear to show functional synapses with ARC GHRH neurons.^18^

Growth hormone is orexigenic in mice, with intracerebroventricular infusion of growth hormone increasing food intake^21^ and genetic upregulation of central growth hormone leading to hyperphagia and obesity.^22^ Growth hormone has been demonstrated to stimulate several orexigenic and anorexigenic neurons of the hypothalamus.^19^ In particular, interactions with orexigenic neurons coexpressing agouti‐related peptide (AgRP) and neuropeptide‐Y (NPY) appear to be important in this effect. Administration of NPY stimulates ARC GHRH and suppresses PeVN somatostatin release, demonstrated in sheep,^23^ and mice.^24^ In rats, intracerebroventricular infusion of NPY suppresses plasma growth hormone, probably through stimulation of somatostatin neurons^25^ indicating an important species difference. In humans, patients with acromegaly have higher plasma AgRP levels, positively correlated with plasma growth hormone and IGF1.^26^ This agrees with the wealth of mouse data and supports an orexigenic role for growth hormone in humans.

An intriguing link between the growth axis and preference for high‐protein diet has been made in rats. Centrally administered GHRH increased protein intake in these animals, and this effect was blocked with antibodies against GHRH.^27^, ^28^ In a human genetic association study, a variant in the gene encoding the somatostatin SSTR2 receptor was associated with altered body mass index and difference in protein content in the diet.^29^ It would make sense that growth axis stimulation might lead to preferential amino acid intake to fuel protein synthesis, however more work is required to fully elucidate this hypothesis.

A central role for growth hormone during fasting has been demonstrated in mice with selective knock out of the growth hormone receptor in orexigenic agouti‐related peptide (AgRP) neurons in the hypothalamus.^21^ Loss of growth hormone receptor in these neurons leads to enhanced fat loss during food deprivation and is accompanied by a reduction in expression of both *Npy* and *AgRP* mRNA. When these mice were treated with 2‐deoxyglucose – a potent hyperphagia‐inducing nonmetabolised glucose analogue that mimics glucose deprivation – their hyperphagia response was inhibited. This work implies a role for growth hormone during fasting to signal energy deficit and stimulate orexigenic pathways to drive eating behaviour.

## 4 GHRELIN AMPLIFIES PULSATILE GROWTH HORMONE SECRETION

The fasting hormone ghrelin was first described in the late 1990s, following the earlier description of its receptor, and synthetic ligands even before that. Ghrelin is released from the stomach and small intestine during fasting, and levels decrease after eating, with its receptors found throughout the hypothalamus.^30^ Ghrelin is considered the only true hunger stimulating hormone, and its main role appears to be in regulating meal‐based eating, at least in part by activating ARC *Npy* neurons.^31^ The name of synthetic ghrelin ligands (growth hormone secretagogues ‐ GHS), and the ghrelin receptor (growth hormone secretagogue receptor – GHSR), are clues to their initially assumed role of the, at the time undescribed growth hormone‐releasing hormone. GHS can stimulate the growth axis both at the level of the pituitary and the hypothalamus to amplify pulsatile growth hormone release.^7^, ^32^ Indeed, ghrelin is thought to inhibit hypoglycaemia through amplification of growth hormone release during fasting.^33^ Despite these different interactions of ghrelin with the growth axis and energy balance, very few of the many mouse models of the ghrelin system have demonstrated the predicted short body length and low bodyweight – or “skinny dwarf” phenotype, as discussed in a recent systematic review.^34^ This may be partially explained by lack of data for somatic growth measures (e.g., body length), and imperfect model systems. However, a recent homozygous *Ghsr‐IRES‐Cre* mouse model from the same authors does show impaired somatic growth, growth hormone secretion and glucose homeostasis.^35^

## 5 LESSONS FROM SEASONAL MAMMALS

The use of seasonal mammals as model species is widespread in neuroendocrinology, and in particular the Siberian hamster (*Phodopus sungorus*, also called the Djungarian hamster) has been well studied to understand seasonal plasticity of energy balance (reviewed in^36^, ^37^). Many species have evolved seasonally variable energy balance phenotypes as an adaptation to a temporally variable environment to conserve energy at a time of low food availability. The time of year is interpreted physiologically by the secretion of melatonin from the pineal gland during the hours of darkness, so that the length of melatonin signal during the night indicates the time of year to the body.^38^ Siberian hamsters under short day length (photoperiod) lose weight in anticipation of a reduction in food availability and can lose up to 40% of their bodyweight over a period of 12–14 weeks. This is so tightly regulated that after a period of food restriction‐induced weight loss, upon refeeding these hamsters will regain weight only to the size appropriate for the length of time they have been in short photoperiod.^39^ The suite of physiological adaptations to short photoperiod, which include reproductive, bodyweight, food intake, pelage and immunological changes are driven largely by the regulation of bioavailability of active thyroid hormone – triiodothyronine (T3) by deiodinase enzymes in tanycyte cells in the hypothalamus.^40^, ^41^ Although this model species is often considered a comparison between lean (short photoperiod /winter) and obese (long photoperiod/summer) phenotypes, the change in bodyweight is composed of both lean and fat mass. These hamsters will typically lose 50% of their previous fat mass, while the majority of weight loss is composed of lean mass.^42^ This seasonal change in organ size has been coined Dehnel's phenomenon and was first described in the common shrew (*Sorex araneus*).^43^

**FIGURE 3:**
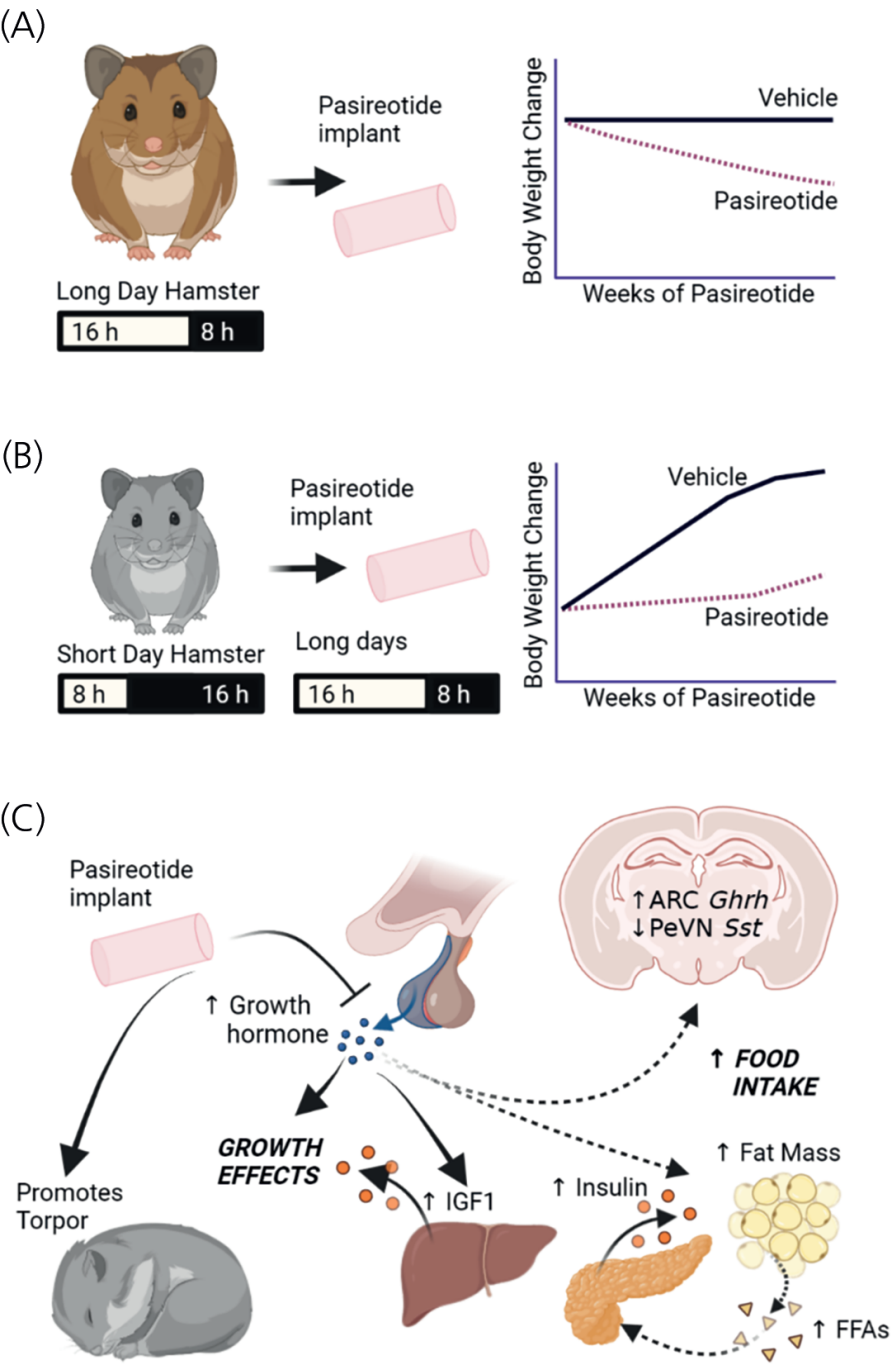
Effects of chronic treatment with somatostatin receptor SSTR5 and SSTR2 agonist pasireotide on photoperiod‐appropriate phenotype. A: Pasireotide treatment causes weight loss in long day Siberian hamsters. B: Pasireotide inhibits photoperiod stimulated weight gain in short day acclimated Siberian hamsters. This demonstrates a role for the growth axis in regulating photoperiod appropriate bodyweight. C: Long photoperiod (long days) probably upregulates this axis through arcuate nucleus (ARC) *Ghrh* (growth hormone‐releasing hormone) and *Sst* (somatostatin) to increase production of growth hormone and/or insulin‐like growth factor‐1 (IGF1). Weight gain and increased organ size is accompanied by increased fat mass and circulating insulin. It has not been tested whether increased insulin is in response to increased growth hormone driven increase in free fatty acids (FFA) or through some other mechanism, but exogenous growth hormone does stimulate this effect. Inhibition of the peripheral growth axis by pasireotide suppresses these effects and promotes likelihood and length of torpor bouts. Suppression of the growth axis by pasireotide is evidenced by altered expression of ARC *Ghrh* and PeVN *Sst* mRNA, probably in response to reduced negative feedback

Seasonal variation in growth hormone and/or IGF1 levels have been reported for many species and in the golden hamster (*Mesocricetus auratus*) growth axis involvement in seasonally appropriate bodyweight was described in the 1990s.^44^ Changes in hypothalamic growth axis components with photoperiod have been demonstrated reproducibly in F344 photoperiodic rats and Siberian hamsters^45^, ^46^; where ARC (but not PeVN) somatostatin expression is highly upregulated in winter or short photoperiod.^47^, ^48^ Using chronic treatment with somatostatin agonist pasireotide, we demonstrated that in Siberian hamsters this change in bodyweight is partially regulated through the growth axis, which acts preferentially at the SSTR5 and SSTR2 somatostatin receptors (Figure 3,^48^). The drug pasireotide was developed to target growth hormone secreting pituitary adenomas and acts largely at the pituitary to suppress growth hormone secretion.^49^ In long photoperiod Siberian hamsters, weight loss was induced by pasireotide, indicating the growth axis is important for maintaining bodyweight in this species and indeed, photoperiod‐induced weight gain was suppressed by pasireotide. Although in long photoperiod pasireotide induced loss of fat mass did not reach significance, in photoperiod driven growth, fat regain was significantly inhibited by this somatostatin agonist. Since this effect goes against the lipolytic action of growth hormone, it suggests additional interactions with the growth axis independent of suppressing growth axis tone.

Interestingly, these studies also identified a role for the somatostatin SSTR5 receptor in the propensity to enter the hypometabolic state of torpor.^48^ Pasireotide treatment in short photoperiod acclimated Siberian hamsters led to a much higher incidence and longer bouts of torpor than either sham or octreotide (a somatostatin agonist with higher affinity for the SSTR2 receptor) treated hamsters. Since both drugs are unlikely to cross the blood brain barrier, it is expected that this is again acting either at the pituitary gland or in metabolically active tissues to suppress metabolism and therefore body temperature.

Together these data support the role of the growth axis in maintenance of body size, and in the regulation of bodyweight. In all the studies where the growth axis was manipulated in Siberian hamsters, the perception of photoperiod remained intact, evidenced by appropriate pelage colour and intact deiodinase expression in the hypothalamus, thus the growth axis effects were downstream of this regulator of seasonal phenotype. Unfortunately, food intake was not measured in these studies, but it is assumed that food intake was probably altered to help bring about whole bodyweight changes.

## 6 THE GROWTH AXIS AND EXERCISE

**FIGURE 4:**
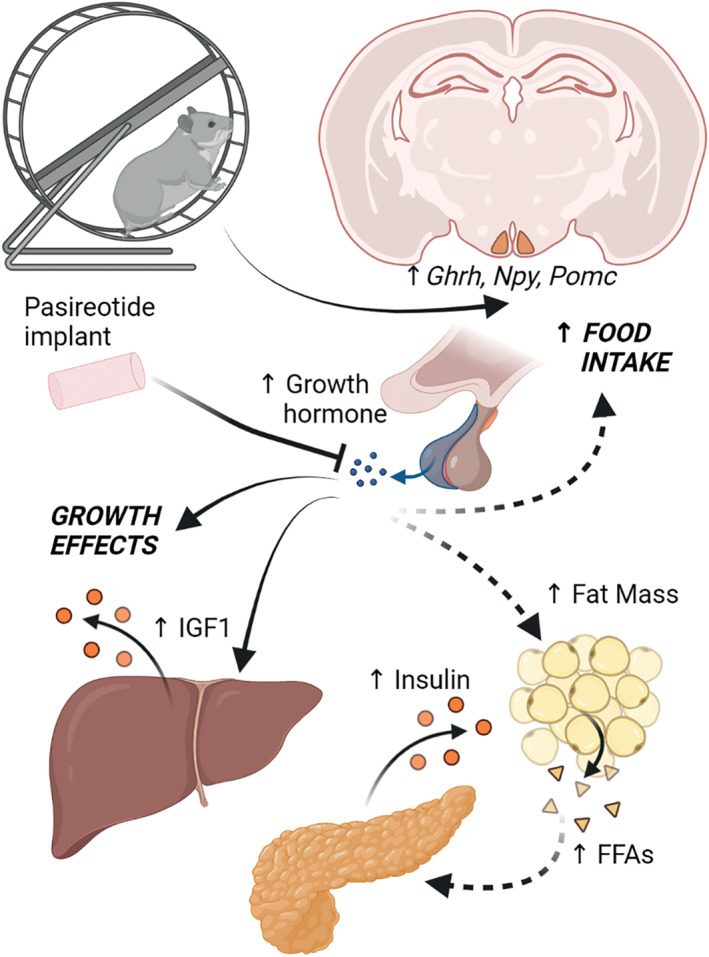
Effects of wheel running exercise in Siberian hamsters. Voluntary wheel running in the normally active phase for short photoperiod or winter acclimated Siberian hamsters leads to weight gain and increased food intake. This is probably driven through stimulation of the growth axis, with raised expression of growth hormone‐releasing hormone mRNA in the arcuate nucleus of the hypothalamus, as well as increased expression of orexigenic *Npy* and anorexigenic *Pomc*. Weight gain and increased organ size is accompanied by increased fat mass and circulating insulin. It has not been tested whether increased insulin is in response to increased growth hormone driven increase in free fatty acids (FFA) or through some other mechanism. The growth and fat mass effects can be inhibited by treatment with long‐acting release somatostatin agonist pasireotide, which is accompanied by reduced serum IGF1 levels. Not to scale

When given access to a running wheel, the Siberian hamster will spontaneously run for long periods of time during their normally active phase (night), and for hamsters housed in long or short photoperiod they will equally run for 10–15 km per day.^50^ Interestingly, this is accompanied with weight gain in short day (or winter) acclimated hamsters,^42^, ^51^ probably through increased food intake. This was first demonstrated in the 1990s in castrated male hamsters.^52^ Wheel running stimulated weight gain is accompanied by increased food intake^42^, ^50^ and while most of the weight gain is from lean mass there is an almost doubling of fat mass in these hamsters, accompanied by increased circulating insulin.^50^ Therefore, this can be considered a poor health response to the wheel running exercise. This effective reversal of Dehnel's phenomenon has been observed in several seasonal rodent species, and upregulation of the growth axis was implicated in a series of experiments by Katarina Borer and colleagues in the Golden hamster in the 1970s.^53^ In our experiments, we recently demonstrated that this exercise stimulated weight gain is associated with increased *Ghrh*, *Npy and Pomc* expression in the ARC in Siberian hamsters.^50^ The ARC *Ghrh* expression was unaltered by somatostatin SSTR5 receptor antagonist pasireotide, indicating action downstream of this change in expression, perhaps unsurprising as it is not predicted to cross the blood–brain barrier. This work is summarised in Figure 4.

Mice lacking growth hormone receptor in leptin receptor‐ (LepR) or steroidogenic factor‐1‐ (SF1) expressing cells in the ventral medial hypothalamus (VMH) have improved aerobic performance with treadmill training.^54^ However, there are discrepancies in exercise response between these mice, depending upon which cells lack the growth hormone receptor. Mice lacking the growth hormone receptor in LepR‐expressing neurons have reduced fat mass and increased skeletal muscle hypertrophy, in contrast to those lacking growth hormone receptors in SP1 neurons which have both increased fat mass and a reduced skeletal muscle size. This intriguing observation implies multiple roles for growth hormone within the hypothalamus, even within a single brain region, and new technologies such as single cell‐, single nucleus‐, and spatial transcriptomics will allow more thorough investigations into these pathways.

While exercise stimulated weight gain might seem counterintuitive, this is probably driven in part by increased food intake. Indeed, in mouse studies, treadmill training stimulates increased activation of NPY/AgRP and tyrosine hydroxylase expressing neurons in the ARC indicated by *cfos* expression, as well as STAT3 phosphorylation in NPY neurons.^55^ This suggests that exercise may drive compensatory eating behaviours through stimulating orexigenic pathways in the hypothalamus. It is certainly tempting to hypothesise that, since neurons expressing these neuropeptides are known to be responsive to growth hormone,^19^ increased food intake may be driven by growth hormone upregulation with exercise training.

Weight gain with exercise is a phenomenon that also occurs in humans, and several studies have demonstrated individual variability and even adverse effects in several health parameters in response to an exercise intervention (reviewed in^56^, ^57^, ^58^), which has also been linked with compensatory eating.^59^ This calls for a more personalised approach in developing exercise‐driven weight loss and health‐improving interventions in human medicine. The interesting exercise response demonstrated by Siberian hamsters makes them an excellent model for human individuals who struggle to lose weight and improve cardiometabolic health through exercise.

## 7 HUMAN GENOMICS AND PERSONALISED MEDICINE

One tool that has become increasingly prevalent in the investigation of individual predisposition to disease is the genome‐wide association study (GWAS; recently reviewed in^60^). This involves the sequencing of the genome of often hundreds of thousands of participants and mapping single nucleotide polymorphisms (SNPs) to health parameters or anthropometric measures. GWAS can be criticised as being difficult to explain mechanistic links since most identified SNPs are in non‐coding regions of the genome. This makes these variants challenging to model in animal systems, since these are subtle changes that are not well conserved cross‐species. This also means that GWAS variants probably do not act by directly altering protein structure, but by epigenetic actions to alter gene expression. These variants are more likely to act in a tissue and environmentally specific manner and may not lead to serious disease state from an early age. However, for many complex diseases (such as obesity and type 2 diabetes), there are only a few rare known monogenic causes, and for most people disease risk is influenced by several different genetic and environmental risk factors. Therefore, lessons from large genetic studies are important to inform our understanding of complex disease.

In addition to the SSTR2 association previously discussed in relation to body mass index and dietary choices,^29^ a recent GWAS in a Swedish population identified two non‐coding SNPs associated with altered fasting growth hormone levels. The first being intergenic in chromosome 17 associated with raised fasting growth hormone, and the second was intronic in growth hormone receptor and associated with decreased fasting growth hormone.^61^ High fasting growth hormone levels are associated with cardiovascular disease risk, and the authors of the study suggest that fasting growth hormone can be a predictor of future disease.^62^ Although the mechanism by which these non‐coding variants may exert their effects is yet to be elucidated, these are two examples by which subtle differences in the genome may alter individual health and risk factors for disease through the growth axis.

Regulatory networks more subtly altering growth axis tone may also play a role in individual variation in growth and energy balance. For example, a protein altering variant in the gene encoding zinc finger homeobox – 3 (ZFHX3) has recently been associated with lower body mass index.^63^ While this transcriptional regulator has not previously been associated with bodyweight, it has been shown to regulate expression of hypothalamic somatostatin mRNA in mice.^64^ Therefore, it is possible that genomic regulation of the growth axis may occur in more subtle and individually variable ways than have previously been accounted for.

## 8 CONCLUSION

The growth axis has been a target for medical research for almost a century, since first isolated from the bovine pituitary and investigation of spontaneous mouse mutants began in the 1920s. The role of the hormones of the growth axis in maintaining growth and healthy glucose homeostasis have been well studied and despite this the subtleties of their central and peripheral roles are still being elucidated. New insights from genetically altered rodent models have advanced our understanding of the different roles of IGF1 and growth hormone to influence growth and energy metabolism as well as indicating interactions in the central regulation of energy balance. The relatively recent discovery of ghrelin has been an important step for the understanding of meal‐patterned eating, and interactions with the growth axis are clear and profound. Questions remain regarding this interaction, and this may be due to an absence of growth‐focussed measurements in earlier studies, therefore highlighting an important lesson in how growth and bodyweight models should be investigated. The orexigenic action of growth hormone through hypothalamic, and especially ARC neurons have been highlighted in recent mouse genetic investigations and this is supported by investigations in the Siberian hamster. ARC SST and GHRH neurons appear to be an important mediator of both seasonal and exercise driven weight gain in the Siberian hamster, and this adds an important step to the pathways explaining photoperiod appropriate growth in this model species. This in turn supports a role for these neurons in appetite regulation in general, but also as a mediator of exercise stimulated compensatory eating. What remains to be shown are specific mechanisms by which these pathways may be stimulated and manipulated. These models, as well as the advent of new technologies to investigate the spatial transcriptome and edit the genome more efficiently, may allow for greater insights to these pathways to combat obesity and metabolic disease. Employing these tools in the context of increased information about the variability of the human genome will be important in informing personalised medicine in the future.

## CONFLICT OF INTEREST

The author declared that they have no conflict of interest to this work.

### PEER REVIEW

The peer review history for this article is available at https://publons.com/publon/10.1111/jne.13133.

## Data Availability

Data sharing is not applicable to this article as no new data were created or analyzed in this study.
